# Neuroprotective effects of the fractions of *Ocimum basilicum* in seizures induced by pentylenetetrazole in mice

**DOI:** 10.22038/AJP.2022.20470

**Published:** 2022

**Authors:** Somaieh Mansouri, Mahmoud Hosseini, Fatemeh Alipour, Farimah Beheshti, Hassan Rakhshandeh, Abbas Mohammadipour, Akbar Anaeigoudari, Mohammad Jalili-nik, Mohammad Reza Khazdair, Amirali Jahani

**Affiliations:** 1 *Pharmacological Research Center of Medicinal Plants, Mashhad University of Medical Sciences, Mashhad, Iran*; 2 *Department of Anatomy, School of Medicine, North Khorasan University of Medical Sciences, Bojnurd, Iran*; 3 *Division of Neurocognitive Sciences, Psychiatry and Behavioral Sciences Research Center, Mashhad University of Medical Sciences, Mashhad, Iran *; 4 *Applied Biomedical Research Center, Mashhad University of Medical Sciences, Mashhad, Iran *; 5 *Department of Anatomy, Faculty of Medicine, Mashhad University of Medical Sciences, Mashhad, Iran*; 6 *Neuroscience Research Center, Torbat Heydariyeh University of Medical Sciences, Torbat Heydariyeh, Iran*; 7 *Department of Physiology, School of Paramedical Sciences, Torbat Heydariyeh University of Medical Sciences, Torbat Heydariyeh, Iran*; 8 *Department of Physiology,* *School of Medicine, Jiroft University of Medical Sciences, Jiroft, Iran*; 9 *Department of Biochemistry, School of Medicine, Mashhad University of Medical Sciences, Mashhad, Iran*; 10 *Cardiovascular Diseases Research Center, Birjand University of Medical Sciences, Birjand, Iran*; 11 *Department of Laboratory Sciences, School of Paramedical Sciences, Mashhad University of Medical Sciences, Mashhad, Iran*

**Keywords:** Neuroprotective, Ocimum basilicum, Oxidative stress, Pentylenetetrazole, Seizures

## Abstract

**Objective:**

Neuroprotective and antioxidant effects of *Ocimum basilicum (O. basilicum) *against pentylenetetrazole (PTZ)-induced seizures were investigated.

**Materials and Methods:**

Mice were divided as follows: (Group 1) Control, (Group 2) PTZ, (Groups 3-5) 50,100 and 200 mg/kg hydro-ethanolic (HE) extract, and (Groups 6-8) 200 mg/kg ethyl-acetate (EAF), N-hexane (NHF) and water (WF) fractions. Minimal clonic seizures (MCS) and generalized tonic-clonic seizures (GTCS) latencies were measured. Biochemical and histological studies were done.

**Results:**

MCS and GTCS latency in HE groups were longer than the PTZ group (p<0.05 to p<0.001). EAF and NHF prolonged the onset of MCS and GTCS (p<0.001). PTZ increased malondialdehyde (MDA) and dark neuron (DN) production while decreased thiol, catalase (CAT) and superoxide dismutase (SOD) (p<0.05 to p<0.001). Pre-treatment by HE and all fractions of the plant attenuated MDA and DN while increased thiol, CAT and SOD (p<0.01 to p<0.001).

**Conclusion:**

EAF and NHF had anticonvulsant properties. The extract and fractions protected the brain from PTZ-induced oxidative damages and showed neuroprotective effects.

## Introduction

Epilepsy is a disease with severe and abnormal discharges of brain neurons which affects roughly 1% of the world population (Yang et al., 2020 ▶). It can remarkably influence cognitive processes and behaviors in epileptic patients (Yang et al., 2020 ▶). Brain tissues oxidative damage due to excessive production of free radicals, has been mentioned to induce the seizure attacks. Meanwhile, it has been confirmed that prolonged seizures can gradually lead to death of neurons due to the induction of lipid peroxidation, oxidative stress, and DNA damage (Kudin et al., 2002 ▶). Treatment of epileptic seizures is usually difficult due to poor passage of antiepileptic drugs through the blood-brain barrier. Furthermore, the anticonvulsant activities of several natural bioactive compounds found in plants extract have been shown (Gupta and Briyal, 2006 ▶). In our previous studies also, the anticonvulsant effects of a number of plant extract have been reported (Vafaee et al., 2015 ▶; Hosseini et al., 2013 ▶; Karami et al., 2015 ▶). 

*Ocium basilicum* (*O. basilicum*) is one of the well-known medicinal herbs from the Lamiaceae family which has displayed therapeutic effects such as anticancer, antioxidant and neuro-protection effects (Bora et al., 2011 ▶; Flanigan and Niemeyer, 2014 ▶). Administration of *O. basilicum* leaf essential oil and its extract has been found to exert hypnotic and anticonvulsant effects (Shakeri et al., 2019 ▶; Oliveira et al., 2009 ▶; Askari et al., 2016 ▶). In another study, the protective effect of ethyl acetate extract of *O. basilicum* against brain tissues oxidative damage in an ischemia brain model has been reported. This neuro-protective effect of *O. basilicum* was accompanied with improvements of motor performance and short-term memory (Bora et al., 2011 ▶). In a recent study, *O. basilicum* showed an anticonvulsant effect accompanied with the attenuation of oxidative stress in the brain tissues, in mice (Khodabakhshi et al., 2017 ▶). Linalool, as a principal component of *O. basilicum* was shown to suppress discharge of neurons in an experimental model of epilepsy when it was intraperitoneally (i.p.) administered in mice (Sakurada et al., 2009 ▶). Also, linalool has been indicated to improve glutathione content and decrease acrylamide-induced lipid peroxidation in brain tissues of rats (Mehri et al., 2014 ▶). Here, we decided to examine the neuroprotective effects of the fractions of *O. basilicum* in seizures induced by pentylenetetrazole (PTZ) in mice. 

## Materials and Methods

**Chemicals,** **animals and treatments**

PTZ was purchased from Sigma-Aldrich Company (St. Louis, USA). The materials utilized for biochemical and histological studies were obtained from Merck Company (Germany). 

Ninety-six of BALB/c male mice (27 ± 3 g) were supplied from Animal Center of Mashhad University of Medical Sciences and kept in controlled experimental conditions. The inclusion criteria were: weight between 24- 30 g, male and age of 9-10 weeks. The mice were randomized into 8 groups (*n *= 12 mice / group): (group 1) Control, (group 2) PTZ (100 mg/kg), (groups 3-5) 50,100 and 200 mg/ kg hydro-ethanolic (HE) extract of the plant (HE 50, HE 100 and HE 200), and (groups 6-8) 200 mg/ kg of N-hexane (NHF), ethyl acetate (EAF) and water (WF) fractions. Groups 2-5 were pretreated with the HE during 3 days before the day when seizure was induced by PTZ (100 mg/kg) (Asgharzadeh1 et al., 2020 ▶). The doses of the extract were selected according to the previous studies (Khodabakhshi et al., 2017 ▶, Bora et al., 2011 ▶, Shakeri et al., 2019 ▶). Groups 6-8 were pretreated with 200 mg/kg of the fractions. This dose was equal to the highest dose of the extract. All injections were accomplished i.p. and the volume of injection was 10 ml/kg. Finally, the brains of the mice were collected and used for biochemical (n=8 from each group) and histological (n=4 from each group) measurements. Animal handling was fulfilled based on the instruction of the Ethical Committee at Mashhad University of Medical Sciences (IR.MUMS.REC.1396.57).

**Preparation of the extract**

The aerial parts including stems, leaves and twigs of *O. basilicum* were mustered from Mashhad area (Razavi Khorasan Province, Iran, 2017). The confirmation of plant was carried out by a botanist (Herbarium number: 12937) and was deposited at the herbarium of School of Pharmacy, Mashhad University of Medical Sciences, Iran. To prepare the HE extracts, the dried plant materials (50 g) were mixed with 300 ml ethanol: water (70:30, v/v) and a Soxhalet apparatus was used to prepare the extract. The moisture of the extract was removed by a vacuum evaporator (Askari et al., 2016 ▶).

To prepare the fractions, the dried hydro-ethanolic extract was mixed with distilled water in a separator funnel. Then, by solvent-solvent extraction, it was fractionated with ethyl acetate and N-hexane. The EAF and NHF were separated to obtain WF (Askari et al., 2016 ▶). 

**PTZ-induced seizures**

Induction of seizure was achieved by PTZ (100 mg/kg). After administrating PTZ, the mice were released in a Plexiglas arena (30 × 30 × 30 cm). Then, animals behaviors were recorded for 60 min (Ebrahimzadeh Bideskan et al., 2011 ▶, Hosseini et al., 2013 ▶, Hosseini et al., 2009 ▶, Hosseini et al., 2011 ▶, Hosseini et al., 2014 ▶) using a camera and by a person who was blinded to the treatments. Delay in onset of minimal clonic seizure (MCS) and generalized tonic-clonic seizure (GTCS) was recorded. 

**Biochemical assessment**

For evaluation of biochemical indicators, the mice were sacrificed and their hippocampus was separated and weighed. Then, hippocampus tissue was homogenized using phosphate buffer (100 mM, pH 7.4). In the next step, homogenized tissue was centrifuged (10000 rpm) for 20 min. In the last step, supernatant liquid was collected and used. 

For determination of total thiol content, 2-nitrobenzoic acid (DTNB) was used. Combination of DTNB with total thiol groups results in 2-nitro-5-thiobenzoic acid (TNB) production which has a peak absorbance at 412 nm. Then, 25 µl of DTNB, 50 µl of water and 5 µl of specimens were shuffled. Ultimately, absorbance was read at 412 nm (Karami et al., 2015 ▶). 

MDA concentration was estimated using thiobarbituric acid (TBA). For MDA level determination, 1 ml of samples was mixed with 2 ml of TBA/trichloroacetic acid (TCA)/hydrochloric acid). In the next step, the mixture was heated for 40 min. Then, the mixture was cooled and centrifuged. Ultimately, the absorbance was checked at 532 nm (Karami et al., 2015 ▶). Superoxide dismutase (SOD) activity in the hippocampus of mice was assessed by method of Madesh and Balasubramanian. Based on this method, the superoxide resulted from auto-oxidation of pyrogallol causes the reduction of tetrazolium to colored formazan with absorbance 560 nm (Madesh and Balasubramanian, 1998 ▶). Measurement of catalase (CAT) activity was done based on dissociation of hydrogen peroxide into water and oxygen. Potassium phosphate, hydrogen peroxide and samples were mixed. Then the absorbance was recorded at 240 nm (Aebi, 1984 ▶).

**Histological studies**

The brains were fixed using a formalin 10% solution. The brains were then embedded in paraffin to provide paraffin blocks. The serial sections of 5-μm thickness were provided from the blocks. Ten sections containing the hippocampus were randomly selected form each block. The sections were then stained using toluidine blue method. A×40 objective lens and a light microscope (UPlanFI, Japan) connected to a computer was used to get photographs from the sections. A 400 mm^2^ frame was used to count the dark neurons in the images transferred to the computer. The number of dark neurons per unit in different regions of the hippocampus including CA1, CA2, CA3 and DG were computed using the following formula: 

NA=∑Q®af.∑P

In this equation “$\sum \overset{\text{®}}{Q}$“ illustrates the sum of counted cell in the areas, "a/f" reveals the area linked to each frame, "ΣP" exhibits the sum of frame referred to points hitting space (Karimzadeh et al., 2012 ▶). 

**Statistical analysis**

The results are displayed as mean± standard error of mean. Analysis of the data was done using one-way ANOVA followed by Tukey's *post hoc* comparison test. A p<0.05 was regarded as statistically significant.

## Results

**Behavioral results**

**Figure 1:**
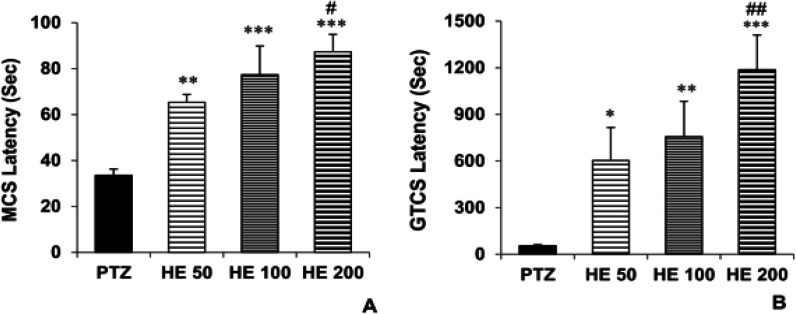
The effects of *O. basilicum* hydro-ethanolic extract (50, 100 and 200 mg/kg) on the minimal clonic seizures (MCS) (A) and generalized tonic–clonic seizures (GTCS) latencies (B). Data is reported as Mean± SEM. *p<0.05, **p<0.01 and ***p<0.001 compared to the PTZ group, #p<0.05 and ## p<0.01 compared to the HE 50 group. PTZ: Pentylenetetrazole, HE 50: Hydro-ethanolic extract 50 mg/kg, HE 100: Hydro-ethanolic extract 100 mg/kg, HE 200: Hydro-ethanolic extract 200 mg/kg

The results showed that all three doses of the HE extract of *O. basilicum* delayed the onset of MCS (p<0.01, p<0.001 and p<0.001 for 50, 100 and 200 mg/kg of the extract, respectively) and GTCS (p<0.05, p<0.01 and p<0.001 for 50, 100 and 200 mg/kg of the extract, respectively) compared to the PTZ group (Figure 1A and 1B, respectively). 

**Figure 2:**
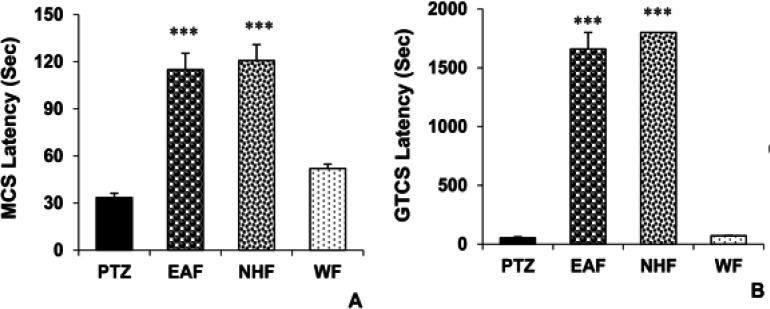
The effects of 200 mg/kg of EAF, NHF and WF of *O. basilicum* extract on the minimal clonic seizures (MCS) (A) and generalized tonic–clonic seizures (GTCS) latencies (B). Data is reported as Mean± SEM ***p<0.001 compared to the PTZ group. PTZ: Pentylenetetrazole, EAF: Ethyl-acetate fraction, NHF: N-hexane fraction, WF: Water fraction

The results also showed that administration of 200 mg/kg of both EAF and NHF before PTZ, postponed the beginning of MCS and GTCS in the EAF and NHF groups versus the PTZ group (p<0.001, for all cases). The water fraction of the plant extract had no significant effects on MCS and GTCS latencies when compared with the PTZ group (Figure 2A and 2B, respectively).

**Biochemical results**

**Figure 3:**
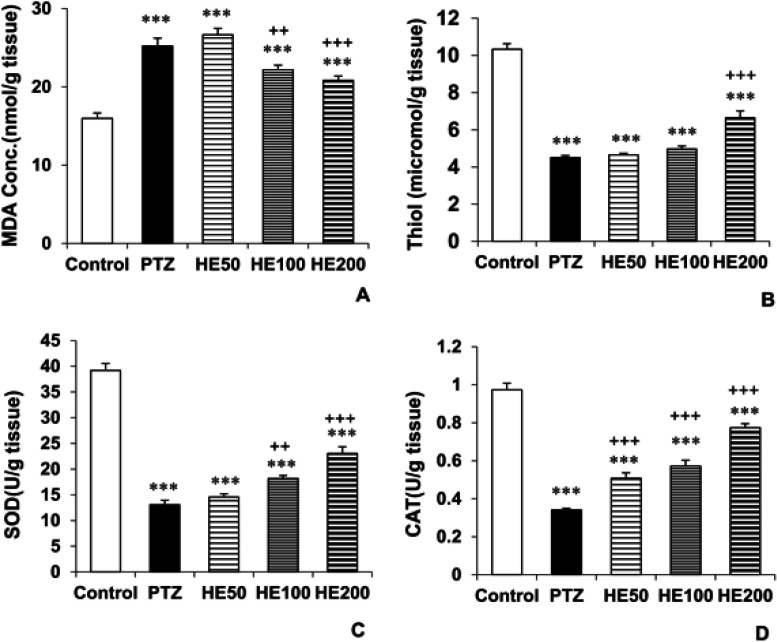
Comparison of the MDA levels (A) and total thiol concentration (B) and SOD (C) and CAT (D) activities in the brain tissue between the control, PTZ and *O. basilicum* extract (50, 100 and 200 mg/kg) treated groups. Data is reported as Mean± SEM. ***p<0.001 compared to the control group. ^++^p<0.01 and ^+++^p<0.001 compared to the PTZ group. PTZ: Pentylenetetrazole, HE 50: Hydro-ethanolic extract 50 mg/kg, HE 100: Hydro-ethanolic extract 100 mg/kg, HE 200: Hydro-ethanolic extract 200 mg/kg

The biochemical analysis of mice brain demonstrated that MDA levels of the PTZ group were significantly higher compared to the control animals (p<0.001). Pretreatment with both 100 and 200 mg/kg of the HE extract reversed the effect of PTZ (p<0.01 and p<0.001 respectively). Injection of 50 mg/kg of the extract did not influence MDA level caused by PTZ administration in the hippocampus of the mice (Figure 3A). The level of MDA in mice hippocampus tissue in the groups treated by 50, 100 and 200 mg HE extract, was higher than the control group (p<0.001 for all doses). In addition, the total thiol groups content in the PTZ group was lower than the control group (p<0.001). Pretreatment with 200 mg/kg of the HE extract of the plant enhanced total thiol group content compared with the PTZ group (p<0.001). The 0 and 100 mg/kg doses of the HE plant extract could not restore PTZ effect (Figure 3B). The total thiol groups content in the groups treated by 50, 100 and 200 mg HE extract was also lower than the control group (p<0.001 for all doses).

Biochemical findings also illustrated a significant decrement in SOD activity of mice hippocampus tissue in the PTZ group with respect to the control group (p<0.001). Treatment with 100 and 200 mg/kg HE extract of the plant increased the activity of SOD in the HE100 and HE200 groups compared to PTZ group (p<0.01 and p<0.001, respectively) (Figure 3C). Hippocampal SOD activity in the groups treated by 50, 100 and 200 mg HE extracts was lower than the control group (p<0.001 for all doses). 

The activity of CAT was lower in the PTZ group compared with the control group (p<0.001). Injection of all three doses of the HE extract resulted in a remarkable increase in hippocampal CAT activity in the HE50, HE100 and HE200 groups compared to the PTZ group (p<0.001 for all cases) (Figure 3D). Hippocampal CAT activity in the groups treated by 50, 100 and 200 mg HE extracts was lower than the control group (p<0.001 for all doses). 

.

**Figure 4:**
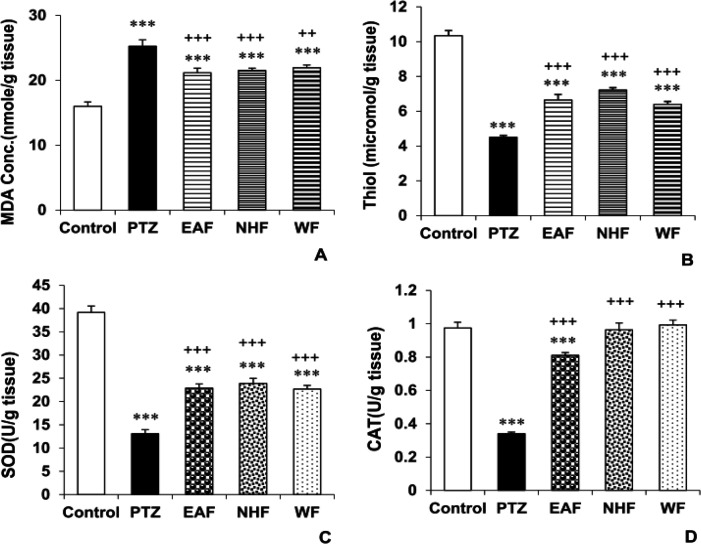
The effects of 200 mg/kg of EAF, NHF and WF of *O. basilicum* extracts on MDA (A) and total thiol concentrations (B) and SOD (C) and CAT (D) activities in mice brain tissue. Data is reported as Mean± SEM. ***p<0.001 compared to the control group. ^++^p<0.01 and ^+++^p<0.001 compared to the PTZ group. PTZ: Pentylenetetrazole, EAF: Ethyl acetate fraction, NHF: N-hexane fraction, WF: Water fraction

**Figure 5:**
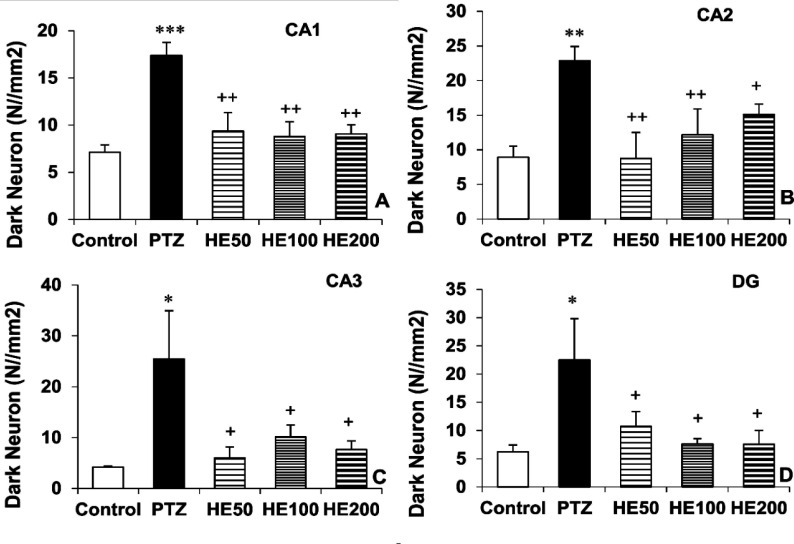
Comparison of the dark neurons in CA1 (A), CA2 (B), CA3 (C) and DG (D) regions of the hippocampus between the Control, PTZ and *O. basilicum* extract (50, 100 and 200 mg/kg) treated groups. Data is reported as Mean± SEM. *p<0.05, **p<0.01 and ***p<0.001 compared to the control group. ^+^p<0.05, ^++^p<0.01 and ^+++^p<0.001 compared to the PTZ group. PTZ: Pentylenetetrazole, HE 50: Hydro-ethanolic extract 50 mg/kg, HE 100: Hydro-ethanolic extract 100 mg/kg, HE 200: Hydro-ethanolic extract 200 mg/kg

In this research we also evaluated the effect of different fractions of *O. basilicum* on biochemical parameters in mice brain. As shown in Figure 5, all three fractions of the plant including EAF, NHF and WF lowered MDA concentration compared with the PTZ group (p<0.001, p<0.001 and p<0.01, respectively) (Figure 4A). According to the results, the total thiol concentration in the EAF, NHF and WF groups was higher than the PTZ group (p<0.001 for all cases) (Figure 4B). In addition, MDA level in the hippocampus of the groups treated by EAF, NHF and WF was higher than the control group (p<0.001 for all cases). Total thiol content in the hippocampus of the groups treated by 200 mg/kg of EAF, NHF and WF was still lower than the control group (p<0.001 for all cases).

As indicated in Figures 4C and 4D, all three fractions of the plant extract enhanced the activity of SOD and CAT in the hippocampal tissues of mice compared to the PTZ group (p<0.001 for all cases). Hippocampal tissue SOD in the groups treated by 200 mg/kg of EAF, NHF and WF was lower than the control group (p<0.001 for all cases). In addition, CAT activity in the hippocampus of groups treated by 200 mg/kg of EAF was lower than the control group (p<0.001)**.**

**Histological results**

**Figure 6:**
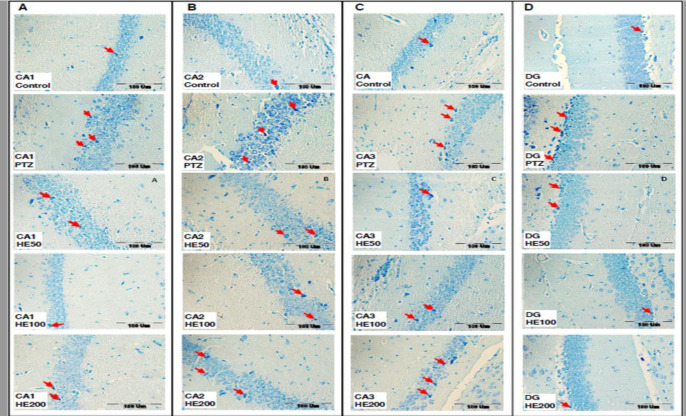
Photomicrograph shows the dark neurons in CA1 (A), CA2 (B), CA3 (C) and DG (D) regions of the hippocampus, toluidine blue stained in the control, PTZ and *O. basilicum* extract (50, 100 and 200 mg/kg) treated groups. Arrow = dark neurons (hyper basophilic neurons), scale bar: 100 µm. PTZ: Pentylenetetrazole, HE 50: hydro-ethanolic extract 50 mg/kg, HE 100: hydro-ethanolic extract 100 mg/kg, HE 200: hydro-ethanolic extract 200 mg/kg

The histological studies (Figures 5A, 5B, 5C and 5D and Figures 6A, 6B, 6C and 6D) showed that PTZ increased the number of dark neurons in areas CA1 (p<0.001), CA2 (p<0.01), CA3 (p<0.05) and DG (p<0.05) of the hippocampus when compared with the control group. Administration of all three doses of the HE extract reduced the number of dark neurons in all four areas of the hippocampus (p<0.05 and p<0.01). There was no significant difference among the three doses of the extract. 

**Figure 7:**
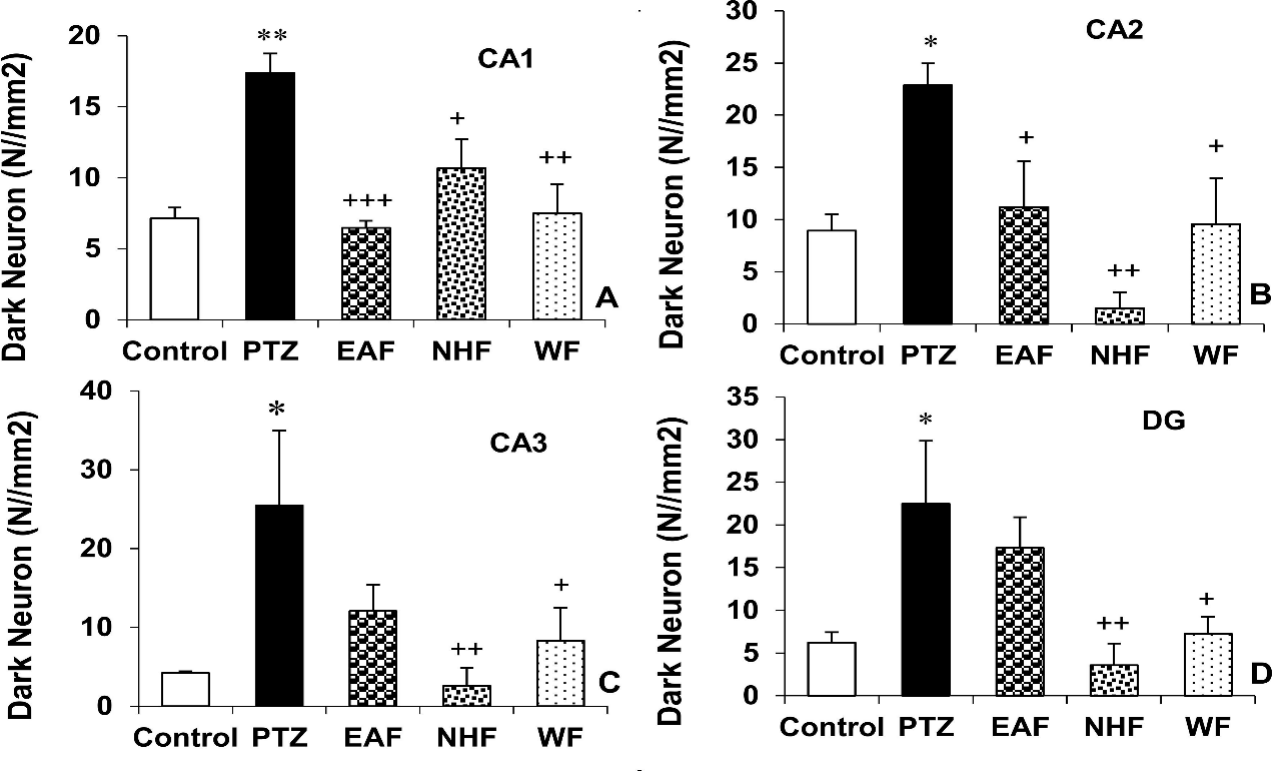
The effects of 200 mg/kg of ethyl acetate fraction (EAF), N-hexane fraction (NHF) and water fraction (WF) of *O. basilicum* extract on dark neurons in CA1 (A), CA2 (B), CA3 (C) and DG (D) regions of the hippocampus of mice. Data is reported as Mean± SEM. *p<0.05 and **P<0.01 compared to the control group. ^+^p<0.05, ^++^p<0.01 and ^+++^p<0.001 compared to PTZ group. PTZ: Pentylenetetrazole, EAF: Ethyl acetate fraction, NHF: N-hexane fraction, WF: Water fraction

**Figure 8:**
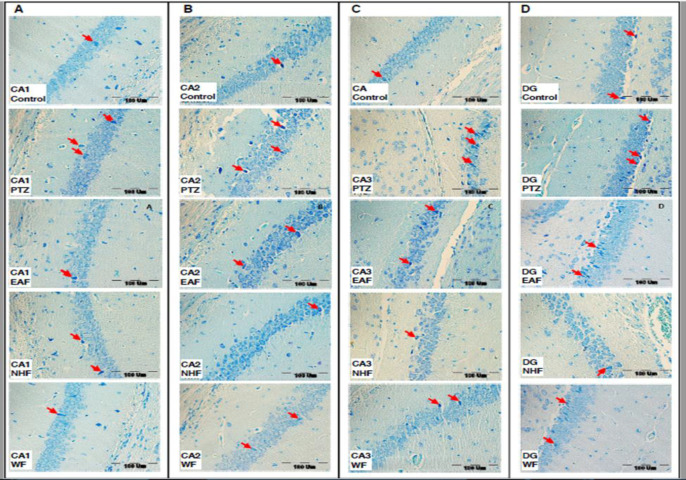
Photomicrograph shows the dark neurons in CA1 (A), CA2 (B), CA3 (C) and DG (D) regions of the hippocampus, toluidine blue stained in the control, PTZ, ethyl acetate fraction (EAF), N-hexane fraction (NHF) and water fraction (WF) groups. Arrow = dark neurons (hyper basophilic neurons), scale bar: 100µm. PTZ: Pentylenetetrazole, EAF: Ethyl acetate fraction, NHF: N-hexane fraction, WF: Water fraction

The results also showed that pretreatment with 200 mg/kg of all three fractions of the plant extract including EAF, NHF and WF decreased the dark neurons in the both CA1(p<0.001, p<0.05 and p<0.01 respectively ) and CA2 (p<0.05, p<0.01 and p<0.05, respectively) regions of the hippocampus compared to the PTZ group. In addition injection of 200 mg/kg of NHF and WF fractions decreased the dark neurons in the CA3 (p<0.01 and p<0.05, respectively) and DG (p<0.01 and p<0.05, respectively) regions of mice hippocampus. EAF of the plant extract was not able to affect dark neurons in the CA3 and DG regions of the hippocampus (Figures 7A, 7B, 7C and 7D, respectively and Figures 8A, 8B, 8C and 8D, respectively). 

## Discussion

The results of the current research revealed that all doses including 5, 100 and 200 mg/ kg of HE of *O. basilicum* and 200 mg/ kg of EAF and NHF but not WF of *O. basilicum* had anticonvulsant properties. It was also shown that the plant extract and their fractions protected the brain tissue from oxidative damages which was presented by a reduced level of MDA and increased levels of thiol, SOD and CAT in the hippocampus. The plant extract and the fractions showed a neuroprotective effect in seizures induced by PTZ which was presented by a decrease in dark neurons in all areas of the hippocampus.

Epilepsy is denoted by abnormal and severe discharges of neurons in the brain which can be associated with muscular rigidity and decreased consciousness (Abdelbary and Fahmy, 2009 ▶). In this work, 

PTZ as a GABA receptor inhibitor (Pagonopoulou et al., 1993 ▶; Gawande et al., 2017 ▶) was used to induce MCS and GTCS in mice. In experimental models, postponement of beginning of MCS and GTCS is considered an indicator of anticonvulsant effect of drugs (Asgharzadeh et al., 2019 ▶). Our data showed that hydro-alcoholic extract of *O. basilicum* delayed the onset of MCS and GTCS in mice. Similarly, we have previously shown that hydro-alcoholic extract of the plant had anticonvulsant effects. In addition, in agreement with the results of the present study, administration of essential oils of *O. basilicum* 60 min before PTZ injection, could prolong the latency time in onset of seizure attacks and lower the intensity of seizures in mice (Koutroumanidou et al. 2013 ▶).

To have an insight about the responsible compounds(s), we here evaluated the fractions of the plant extract and the results showed that the ethyl acetate and N-hexane fractions postponed the onset of MCS and GTCS in mice. Based on our findings, water fraction of *O. basilicum* did not affect the beginning of PTZ- caused MCS and GTCS in mice. Considering these results, it seems that the components responsible for the anticonvulsant effects of the plant are not water soluble and are probably lipophilic compounds. 

The results of the present study showed that PTZ-induced seizures were followed by a decline in thiol, SOD and CAT while hippocampal tissue MDA was increased in epileptic rats. These results confirm an oxidative stress status after seizure attacks (Patel, 2004 ▶). It has been previously reported that epilepsy is followed by neuronal damage especially in the hippocampus (Karimzadeh et al., 2012 ▶). Both neuronal damage and oxidative stress have important roles in cognitive disturbances which are seen in epileptic persons (Ali et al., 2018 ▶). Our results also confirmed that besides oxidative stress, PTZ induced seizure terminated to an increased level of dark neuron production in the hippocampus. 

Considering the neuronal damage which occurs following seizure attacks and considering the fact that oxidative stress has an important role in the pathogenesis epilepsy and seizure and also act as a main contributor in the complications of seizure, it seems that the anti-oxidant compounds are very useful in the treatment of epilepsy. 

Based on the data of the present study, hydro-alcoholic extract of *O. basilicum* also balanced the oxidative stress status in the hippocampus of the mice. The results showed that the hydro-ethanolic extract of the plant decreased hippocampal MDA while improved thiol, SOD and CAT. Interestingly, the hydro-ethanolic extract of the plant reduced the dark neurons in all sub-region areas including CA, CA2, CA3 and DG. Considering these results, it seems the *O. basilicum* hydro- ethanolic extract has protective effects against brain tissues oxidative damage and subsequent neuronal damage which occurs after seizures. 

In this study, we also tested the effects of different fractions of *O. basilicum* on oxidative state of mice hippocampus following PTZ injection. We figured out that all fractions including ethyl acetate, N-hexane and water fractions attenuated MDA while increased SOD, CAT and thiols. The fractions also prevented from dark neuron production in the hippocampus. Therefore, it seems that all three fractions have anti-oxidant and neuroprotective effects (Asgharzadeh et al., 2020 ▶, Mansouri et al., 2021 ▶).

Since the water fraction of the hydro-ethanolic extract of *O. basilicum* showed anti-oxidant effects, it was recommended as a responsible mechanism for anticonvulsant and neuroprotective effects of the fraction however, it needs more investigations in future studies.

The precise mechanism(s) and compounds responsible for beneficial effects of the plant were not evaluated in the present study and it needs to be investigated in the future but presence of several ingredients in* O. basilicum *extract including estragole, linalool, eugenol, anthocyanin, flavonoids and phenols has been confirmed (El-Soud et al., 2015 ▶). Researchers have proven that linalool as a monoterpene compound had beneficial effects against high blood pressure (Anjos et al., 2013 ▶), inflammatory reactions and generation of oxidant agents in rats (Huo et al., 2013 ▶). This basic substance of *O. basilicum* extract has been also shown to prevent memory disturbance in mice (Lee et al., 2018 ▶) which may have a role in beneficial effects of the plant seen in the current research. 

The modulating effect of monoterpenes on glutaminergic and GABAergic transmission has been also documented (Szabadics and Erdelyi, 2000 ▶). The anticonvulsant activity of linalool against PTZ-induced convulsion has been reported (Elisabetsky et al., 1999 ▶). In addition scientific findings showed that linalool in *O. basilicum* extract can cause anticonvulsant effects through the inhibition of glutaminergic neurons (Brum et al., 2001 ▶) and suppression of voltage-gated currents (Narusuye et al., 2005 ▶). Therefore, we suggest that a part of anticonvulsant effects of different fractions of *O. basilicum *that was seen in the present study may be mediated by effects on brain neurotransmitters. 

Finally, the limitations of the current study were that the exact compounds responsible for anticonvulsant, anti-oxidant and neuroprotective effects of *O. basilicum* were not determined. In addition, the exact mechanism(s) for beneficial effects of the plant and the fractions need to be more investigated in future studies. The present data demonstrated that the hydro-alcoholic extract of *O. basilicum* aerial parts and its different fractions modulated PTZ-stimulated seizures in mice and also showed neuroprotective effects. Based on our findings, anticonvulsant and neuroprotective properties of *O. basilicum* extract and its fractions were accompanied with reduction in oxidative damage in the mice brain tissues. Further studies need to be done to determine the responsible compounds and mechanisms.

## Conflicts of interest

The authors have declared that there is no conflict of interest.
